# Smartphone-Based Inertial Odometry for Blind Walkers

**DOI:** 10.3390/s21124033

**Published:** 2021-06-11

**Authors:** Peng Ren, Fatemeh Elyasi, Roberto Manduchi

**Affiliations:** Computer Science and Engineering, UC Santa Cruz, Santa Cruz, CA 95064, USA; felyasi@ucsc.edu (F.E.); manduchi@soe.ucsc.edu (R.M.)

**Keywords:** inertial odometry, wayfinding, indoor pedestrian tracking

## Abstract

Pedestrian tracking systems implemented in regular smartphones may provide a convenient mechanism for wayfinding and backtracking for people who are blind. However, virtually all existing studies only considered sighted participants, whose gait pattern may be different from that of blind walkers using a long cane or a dog guide. In this contribution, we present a comparative assessment of several algorithms using inertial sensors for pedestrian tracking, as applied to data from WeAllWalk, the only published inertial sensor dataset collected indoors from blind walkers. We consider two situations of interest. In the first situation, a map of the building is not available, in which case we assume that users walk in a network of corridors intersecting at 45° or 90°. We propose a new two-stage turn detector that, combined with an LSTM-based step counter, can robustly reconstruct the path traversed. We compare this with RoNIN, a state-of-the-art algorithm based on deep learning. In the second situation, a map is available, which provides a strong prior on the possible trajectories. For these situations, we experiment with particle filtering, with an additional clustering stage based on mean shift. Our results highlight the importance of training and testing inertial odometry systems for assisted navigation with data from blind walkers.

## 1. Introduction

Smartphone-based odometry systems for pedestrian tracking in indoor, GPS-denied environments have received considerable attention in recent years. These systems may help a person reach a gate in an airport [1] or a shop in a mall [2], navigate a museum [3], or find one’s car in a parking lot [4]. Among the various approaches considered in the literature, technology based on inertial sensors have a number of practical advantages. For example, inertial-based odometry does not require the installation of infrastructure such as Bluetooth low energy (BLE) beacons [5]. In addition, no prior calibration (“fingerprinting”) is necessary, unlike for systems based on Wi-Fi [6] or BLE beacons. Compared with systems that use a camera to determine the user’s location (visual-based odometry [7]), and that, thus, require good unoccluded visibility of the scene, inertial systems are able to track the user even when they keep the phone in their pocket. The downside of this modality is that the user’s location is tracked by integrating inertial data, which leads to possibly large errors due to accumulated drift. A number of strategies to deal with drift have been proposed, including zero-velocity updates [8], spatial constraints (e.g., Bayes filtering using a map of the environment [9]), and machine learning [10]. Multiple well-calibrated inertial datasets (containing data from accelerometer and gyros) collected from regular smartphones carried by human walkers have been made available in recent years [10,11,12].

Among the communities of potential users of this technology, blind travelers arguably stand to benefit the most. Wayfinding can be extremely challenging for those without sight, who do not have access to landmarks and other visual information. Blind individuals can easily get lost in unfamiliar environments, which may discourage independent travel for these individuals. Reliable pedestrian tracking systems could improve safety and confidence for blind people navigating a shopping mall, a hospital, or a transit hub.

Although, in principle, the same mechanisms designed for sighted people could also be used by blind travelers, it is well known that the gait characteristics of blind individuals using a long (white) cane or a dog guide are different from those of sighted walkers [13,14,15,16]. For example, use of a long cane (e.g., using the two-point touch technique [17]) may result in large side-to-side swings. When “shorelining” a wall to maintain a straight trajectory, vibrations from the cane hitting the wall may be picked up by the smartphone’s accelerometer. Walking without sight often leads to bumping onto obstacles or even onto people, requiring one to stop and re-orient themselves. These events (combined with other situations, such as negotiating a doorway or opening a door) contribute spurious inertial measurements that could challenge odometry algorithms designed for “cleaner” data associated with sight-assisted walking. Hence, it is important to evaluate these systems on “real” data from blind walkers. In this contribution, we use the WeAllWalk dataset [16], which is the only publicly available collection of inertial data from blind individuals. WeAllWalk contains inertial data from 10 blind participants, each carrying two iPhones, who traversed a number of paths in two different buildings using a long cane or a dog guide as travel aid.

We consider two realistic situations in this work. In one situation (*map-less*), no map of the environment is available. Even without a map, pedestrian tracking could be useful for *assisted return*: In this case, the system can provide spatial information and direction to a blind user who is backtracking their path. [18,19,20,21]. In the second situation (*map-assisted*), we assume that a map of the place is available in digital form. Clearly, this is the most desirable case, as it enables the system to provide turn-by-turn directions to the desired destination. In addition, a map provides a strong constraint on the reconstructed trajectory (e.g., the trajectory cannot go through a wall). In both cases, we consider two different types of pedestrian dead-reckoning (PDR) systems: a simple PDR using an RNN-based step counter, coupled with (possibly drift-corrupted) heading information as provided by the iPhone’s sensors; and a more sophisticated system (RoNIN [10]), based on deep learning, that was shown to produce state-of-the-art results when tested with existing inertial datasets. Since RoNIN was trained with sighted walkers, we experimented with fine-tuning its network with data from blind walkers in WeAllWalk. For the *map-less* case, in which the strong constraint of wall impassibility cannot be relied on, we experimented with a simple turn/segment path representation. This is appropriate for buildings with corridors that intersect at discrete turning angles at multiples of 45° or 90°. Besides providing a strong spatial prior which can be used to compensate for drift, this representation is particularly useful for the verbal description of a path [18]. As an example, a path could be represented as “walk straight for 80 steps, then make a right turn, walk straight for 50 more steps, make a left turn, then after 40 steps you will reach your destination”. It is important to note that for a turn/segment representation to be successful, turns must be detected robustly, which may be challenging in some situations. For example, a blind walker may stop and turn around to get their bearings, or to listen to a sound that may help with orientation, something that could mistakenly be interpreted by the system as a path turn [18]. Blind walkers (especially those who do not use a dog guide) often tend to veer when attempting to walk on a straight line, and this unwanted veering may generate false turn detections. We introduce a two-stage turn detection system, formed by an orientation tracker and a straight walking detector, that proved reliable in the face of such adversarial situations. For the *map-assisted* case, we integrate the two considered systems with a standard particle filter, which was enhanced with a module that finds the mode of the posterior state distribution.

These are the main contributions of this work:

- We introduce a new mechanism (based on a recurrent neural network) to robustly identify when a user is walking regularly along a straight path;
- We propose a new algorithm for turn detection that is unaffected by drift. This algorithm finds the difference in user’s orientation between the beginning of a “straight walking” segment, and the end of the prior segment. Orientation drift is tracked by a Mixture Kalman Filter;
- We describe a new method to identify the modes of the posterior probability distribution of the user’s location tracked by a Particle Filter;
- We study the performance of different mechanisms of path reconstruction (map-less and map-assisted), as well as of an LSTM-based step counter of our turn detector, when applied to the data collected from the blind walkers represented in the WeAllWalk dataset. This analysis was conducted using a variety of assessment metrics. Our results highlight the importance of training these algorithms with data from the same community of users (in this case, blind walkers) these systems are designed for.

This article is organized as follows. We first review the related work (including the WeAllWalk dataset) in Section 2. The step counting, turn detection, and path reconstruction algorithms used in this work are presented in Section 3. Experiments are described in Section 4, where we also introduce the considered training and test modalities. These experimental results are then discussed in Section 5. Section 6 has the conclusions.

## 2. Related Works

### 2.1. Pedestrian Dead Reckoning (PDR)

Perhaps the simplest method to track the location of a walker is to count steps while measuring the user’s orientation at all times [22,23,24,25,26,27,28]. Step counting is traditionally performed by finding peaks or other features in acceleration or rotation rate signals (e.g., [29,30,31]). More recently, recurrent neural networks (RNN) have been proposed as a robust alternative to “hand-crafted” algorithms [32,33,34].

The orientation of the phone can be obtained by proper integration of the data from the accelerometer and gyro [35,36], but this typically results in accumulated drift. Turns can be detected, for example, by measuring short-time variations of the azimuth angle (or of the rotation rate from the gyro [37]), which are unaffected by slowly varying drift. Flores et al. [38] proposed a system based on dynamic programming to estimate the discrete walker’s orientation along with drift. Although effective in the tests of [38] with sighted walkers, this algorithm gave poor results with blind walkers [16]. Another problem is how to decouple the orientation of the phone from the direction of walking. A number of algorithms for the estimation of the direction of walking, independently of the orientation of the phone, have been developed [39,40,41,42]. Another topic of interest is the robust detection of steps and of stride lengths, which are used as a proxy for the walker’s velocity [29,30,31], [43,44,45,46,47,48]. When a map of the environment is available, it may provide a strong constraint for the space of possible trajectories. Bayes filtering (in particular, particle filtering [9]) is normally used in these situations [49,50,51,52].

### 2.2. Learning-Based Odometry

In recent years, a number of data-driven techniques for odometry, that rely less on models and more on machine learning, have emerged. For example, RIDI [11] regresses user velocity from the time series of linear accelerations and angular velocities. IONet [53] uses a deep neural network to compute user velocity and heading. RoNIN [10] processes inertial data in a heading-agnostic reference frame using a variety of deep network architectures. Importantly, RoNIN is able to decouple the phone’s orientation from the user’s orientation when walking. This means that tracking is unaffected by any possible repositioning of the phone (e.g., if the user moves the phone to a different pocket). A number of other learning-based algorithms for computing the walker’s velocity, or for detecting steps and measuring stride lengths, have been recently proposed [33,34,54,55,56,57,58,59,60,61].

### 2.3. Inertial Navigation for Blind Travelers

Wayfinding systems for blind individuals have received special attention, given the potential of this technology to enable independent travel. Although GPS can be used for localization in the outdoors, indoor environments (office buildings, shopping malls, transit hubs) can be particularly daunting for blind travelers. By tracking the location of the blind walker, a wayfinding system can provide turn-by-turn directions, present location-based information (e.g., announce the presence of a store nearby), or help users re-trace their path from the starting point. Several wayfinding techniques specifically designed for blind travelers [62] have been considered, including visual odometry (e.g., [63,64]), BLE beacon-assisted navigation [65], and magnetic navigation [66]. Although inertial data are often used to integrate information from other sensors, relatively little research work has explored the use of inertial sensors alone for blind navigation. This includes work by Fallah et al. [67] and Riehle et al. [68]. Flores and Manduchi [18] proposed an inertial system for assisted return (backtracking) that used a turns or steps representation of indoor environments in a study with six blind participants.

### 2.4. Inertial Data Sets

Well-calibrated datasets are important for performance assessment of odometry algorithms, and are essential for the training of learning-based systems. RIDI [11] was built using a smartphone equipped with an RGBD camera, which enables complete pose (location+orientation) reconstruction at each time using Visual SLAM. OxIOD [12] is a data collection with four different phone placements. The walkers’ location was tracked by a Vicon system. RoNIN [10] is a large dataset with inertial data collected from a smartphone. A different phone, equipped with an RGBD camera, was used for ground-truth walker pose estimation. More limited in scope, the dataset presented in [69] contains inertial data from a smartphone with time-stamped heel strike events and stride lengths, as computed by a foot-mounted inertial system.

#### The WeAllWalk Dataset

WeAllWalk (https://datadryad.org/stash/dataset/doi:10.7291/D17P46; accessed on 9 June 2021) [16] contains data collected from ten blind and five sighted participants. Nine of the ten blind walkers used a long cane while walking. Two of them repeated the trials using their dog guide. One participant only used her dog guide. The sighted participants and eight of the blind participants walked along six different routes: T1–T4 in one building, and T5-T6 in a second building. The last two blind participants walked on two long routes (T7–T8 for one participant, T9–T10 for the other participant) in the second building. Cumulatively, participants in WeAllWalk walked for 7 miles. Routes were chosen to have a variety of lengths (from 75 to 300 m) and complexities (including multiple 45°, 90° and 180° turns). In some cases, a turn was preceded or followed by a door that had to be opened. The space where data collection took place was partitioned in rectangles (“straight” or “turn”) defined on the floor maps; this information is also provided with the dataset. Specifically, for the i-th path, a set $\left\{P_{j}^{i}\right\}$ of consecutive segment endpoint locations (*waypoints*) is provided.

Participants carried two smartphones (iPhone 6s), placed in different locations of their choice on their garments. Each smartphone recorded data (sampled at 25 Hz and timestamped) from its tri-axial accelerometers, gyroscopes, and magnetometers, as well as attitude data produced by the iOS Core Motion framework via Apple’s proprietary sensor fusion algorithms. Heel strike times were recorded for each foot using two inertial sensor units clipped to the participants’ shoes. Overall, approximately 20,000 heel strikes were recorded.

The time elapsed from the beginning to the end of each route traversal was divided into contiguous intervals, where each interval corresponds to the participant either walking along a “straight” segment in the path, or walking on a “turn” segment. In addition, the dataset provides time-stamped annotations of *features* (136 overall), defined as particular events such as opening a door, bumping into an obstacle, being caught in a door opening, or stopping momentarily. These events are normally associated with anomalous characteristics in otherwise regular inertial data time series.

## 3. Algorithms

In this section, we describe the individual algorithms used by the considered path reconstruction systems. The inertial data of interest from the phones include: *attitude*, defined as the 3-D orientation with respect to a fixed “world” reference frame with Z axis pointing in the direction of gravity; *gyro*, a 3-D vector with angular velocities; *acceleration*, as measured by the 3 axes of the accelerometer; *user acceleration*, which is a 3-D vector with the actual phone acceleration (i.e., with gravity removed). Note that all of these quantities (except for *gyro* and *acceleration*) are obtained using the device’s proprietary sensor fusion algorithm from data acquired by the onboard accelerometers and gyroscopes. From this data, we also derive *azimuth* (or heading), which is the angle between the Y axis of the phone frame, projected onto the horizontal plane (defined by X and Y axes of the world frame), and the Y axis of the world frame. It is important to note that *attitude* and, thus, *azimuth* are prone to drift, where, for our purposes, drift can be modeled as a slowly varying bias.

### 3.1. Step Counting

We implemented a step counting system based on LSTM [70] (a popular type of RNN). Prior work (e.g., [32]) used a bi-directional LSTM, which can increase robustness by considering a whole batch of data at once. This approach would not be appropriate for wayfinding or assisted return application, where timely step detection is necessary (e.g., to constantly track the position of the walker in the route.) We, thus, only considered a regular, uni-directional LSTM for our application. Our LSTM takes in input user acceleration (over 3 axes) and rotation rate (over 3 axes). These vectors are pre-multiplied by the inverse of the attitude matrix, in order to obtain a heading-agnostic reference frame [10]. The data for each axis are normalized to zero mean and unit variance. The LSTM is trained to produce a sequence of values that are close to the desired output, represented by a sequence that is uniformly equal to 0 except for the times of occurrence of a heel strike, when it is set to 1 (more precisely, we transform each impulse into a narrow triangular wave with length of three samples). The LSTM is trained with Keras using 100-samples windows (4 s), and least squares loss. Note that the output of LSTM is a sequence of numbers between 0 and 1, and is transformed to a binary signal by applying a suitable threshold S. For each sequence of consecutive LSTM output samples that exceed this threshold, we select the midpoint to be the time of estimated heel strike. Our LSTM uses a 2-layer network with hidden unit size of 6. We found, through initial experiments, that the network was deep enough for the task, and adding more layers would increase the risk of overfitting. We used the Adam optimizer and dropout for regularization. Training is performed with batch size of 256 and initial learning rate of 0.005 (decreased by a factor of 2 after 50 epochs) over a total of 64 epochs.

**Figure 1:**
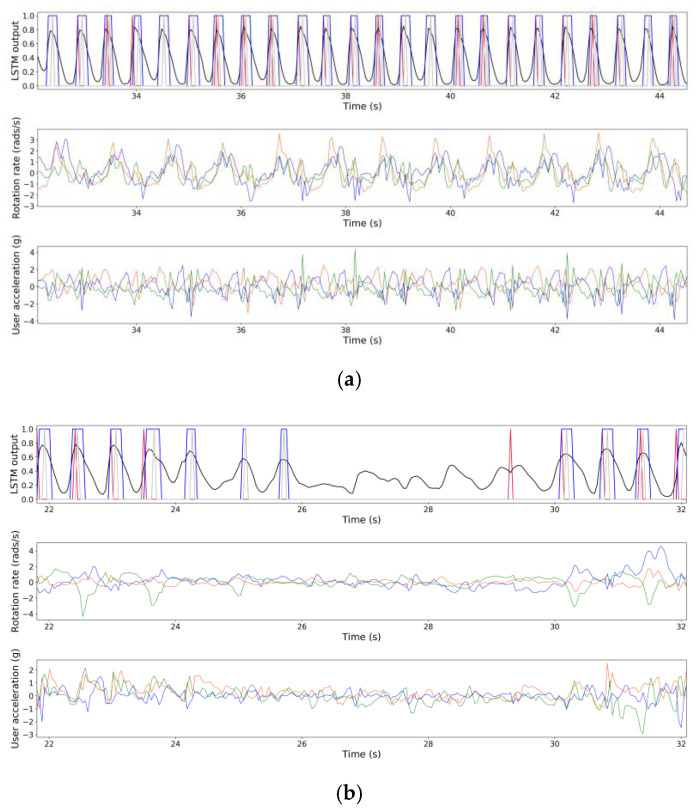
Examples of step detection. (**a**) Top row: the output of LSTM (black line) is thresholded, and the midpoints of the resulting positive segments (gray line) are taken as the estimated times of heel strike (ground-truth shown by red line). The LSTM takes in input the 3-axes rotation rate (middle row) and the 3-axes user acceleration (bottom row). Examples of overcounts are seen in (**b**) between t = 24 s and t = 26 s. An example of undercount is seen between t = 29 s and t = 30 s.

Examples of step detection are shown in Figure 1. Although our step counter works robustly in most conditions, we noticed that the LSTM output exhibits a decaying oscillatory behavior when one stops walking. In addition, sometimes the first one or two steps may be missed when one starts walking.

#### 3.1.1. Stride Length

In order to use step counting for odometry, one needs to define the walker’s stride length. A number of techniques have been proposed for stride length estimation from inertial data [46,47,48], including recent approaches using deep networks [33,34]. We experimented with several of these algorithms, but failed to obtain consistent and accurate results. Thus, we decided instead to use a fixed stride length SL, regressed from the known lengths of the paths traversed in WeAllWalk, and the ground-truth number of steps in each path for each participant in the training set.

### 3.2. Two-Stage Turn Detection

**Figure 2:**
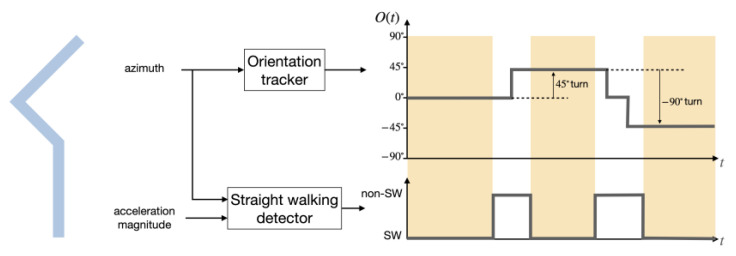
Diagrammatic example of our two-stage turn detector. The blue line (**left**) represents a path taken by a walker, with a 45° (**left**) turn, followed by a −90° (**right**) turn. A turn is detected by comparing the orientation $O\left(t\right)$ produced by the orientation tracker between two consecutive straight walking (SW) segments (highlighted in yellow).

The trajectory of a walker in an environment characterized by a network of straight corridors crossing at discrete angles (e.g., multiples of 45° or 90°) can in most cases be described as a sequence alternating straight segments with turns, with discrete turning angles. When designing a turn detector to be used by blind walkers, one needs to consider various situations that may trigger a false detection. For example, the walker may stop and rotate their body to re-orient themselves. Walkers may need to swerve when avoiding a perceived obstacle, or when mistakenly veering off a straight path. In order to reduce the likelihood of false detections, we implemented a simple two-stage procedure for robust turn detection. The system (shown in Figure 2) is comprised of an orientation tracker, which returns the discrete angle of the walker’s orientation in the horizontal plane; and of a straight-walking (SW) detector, which identifies the time periods in which the user is walking on an approximately straight path. The idea is that a turn is not expected to occur during a SW segment; whereas any numbers of turns could be detected (correctly or otherwise) outside such intervals. A turn is declared when the user’s orientation during a SW segment is different than in the previous SW segment.

#### 3.2.1. Straight Walking (SW) Detector

**Figure 3:**
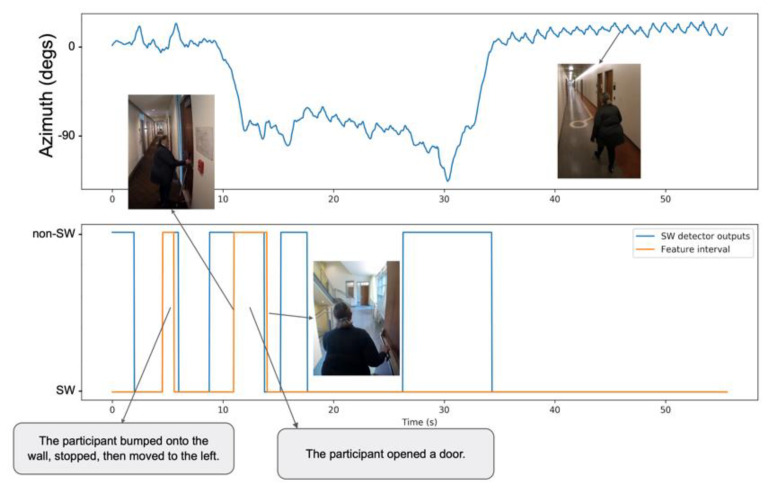
Example of SW segment detection using our GRU system. Top: Azimuth signal; Bottom: output of SW detector (blue line), shown together with the segments marked as “features” in WeAllWalk (orange line).

Our straight walking (SW) detection system is designed to identify time intervals during which the user walks “regularly” on a straight path. SW detection is performed using a GRU, which is a simple type of recurrent neural network [71]. Our GRU processes a pair of signals: the azimuth angle and the user accelerometer magnitude (previously smoothed by a Gaussian filter with σ=15). The GRU is trained on data samples recorded within straight segments (after removing *feature* sub-segments), as these are considered to be representative of SW segments. We manually annotated sub-segments at the beginning and at the end of each trial, when the user was known to be standing still, and removed them from the data labeled as SW. Data from these sub-segments, as well as data from *feature* sub-segments and data from turn segments, are labeled as non-SW. The GRU is tasked with predicting the label value with a delay of approximately 1 s (30 samples). We found this to be an acceptable trade-off between timeliness (which calls for small delay) and the need to see enough forward data to correctly predict the label after a transition. The system was trained using Keras, using 150-samples windows. Other training parameters include: GRU hidden unit size of 32; drop-out rate of 0.4; 3 training epochs, batch size of 2048. An example of SW detection using our GRU system is shown in Figure 3.

#### 3.2.2. Orientation Tracker

The goal of this module is to track the walker’s orientation from azimuth data in the face of slowly changing drift. We assume that the walker is at each time t in one of N fixed orientations $\left\{O^{k}\left(t\right)\;=k\cdot 360{}^{\circ}/N\right\}$, where N = 8 or 4 (orientations at multiples of 45° or 90°, respectively). The sequence of orientations is modeled as a Markov chain. The azimuth measurement, $\theta \left(t\right)$, is assumed to be affected by drift, $d\left(t\right)$, which is modeled as a random walk [72], with additional white Gaussian noise. We use the standard Gauss–Markov hypothesis to write:

(1) $$p\left(\theta \left(t\right)|O\left(t\right),d\left(t\right),\theta \left(1:t-1\right)\right)\;=p\left(\theta \left(t\right)|O\left(t\right),d\left(t\right)\right)\;=N\left(O\left(t\right)+d\left(t\right),\sigma_{\theta }^{2}\right)\;p\left(d\left(t\right)|d\left(t-1\right),\theta \left(1:t-1\right)\right)\;=p\left(d\left(t\right)|d\left(t-1\right)\right)\;=N\left(d\left(t-1\right),\sigma_{d}^{2}\right)\;P)O^{k}\left(t\right)|O^{m}\left(t-1\right),\theta \left(1:t-1\right))=P(O^{k}\left(t\right)|O^{m}\left(t-1\right))=\left\{\begin{array}{cc} q, & m=k\\ \left(1-q\right)/2, & \left|m-k\right|\;=1\\ 0, & \mathrm{otherwise} \end{array}\right.$$

In the equations above, $\theta \left(1:t-1\right)$ represents the sequence of azimuth measurements before time t. $N\left(\mu ,\sigma^{2}\right)$ is a normal distribution, while q represents the prior probability that the user’s orientation does not change in two consecutive time instants. Note that turns $O^{m}\left(t-1\right)\to O^{k}\left(t\right)$ by more than the prescribed interval 360°/N (i.e., $\left|m-k\right|\;>1$) are not allowed in this model. For example, if N=8, a turn by 90° would be tracked as two consecutive turns by 45°.

We use a Mixture Kalman Filter (MKF [73]) to compute the posterior distribution of discrete orientations $P(O^{k}\left(t\right)|\theta \left(1:t\right))$ and of drift $p\left(d\left(t\right)|\theta \left(1:t\right)\right)$ at each time. The MKF algorithm maintains a list of orientation sequences, with a Kalman filter associated with each sequence. At each time instant, a new orientation is added to each sequence by sampling from the posterior distribution $P(O\left(t\right)|\theta \left(1:t\right))$. Our MKF system maintains a set of 50 Kalman filters, which go through a standard process of resampling and rejuvenation [73]. The walker’s orientation at time *t* is taken to be the maximizer of $P(O\left(t\right)|\theta \left(1:t\right))$. The parameters $\sigma_{\theta },\;\;\sigma_{d}$, and q are learned from the training data by finding (via grid search) the minimum of the weighted sum of overcount and undercount rates of the two-stage turn detector, as defined later in Section 4.3. Note that we give a larger weight (equal to 2.5) to the undercount rate, because we noted that turn undercounts often affect the reconstructed path more than turn overcounts, possibly because two consecutive incorrectly detected turns with opposite angles compensate for each other.

#### 3.2.3. Turn Detection

In our two-stage system, turn events are detected when the discrete walker’s orientation $O\left(t\right)$ in a SW segment is different from that in the previous SW segment. Note that the orientation of a walker is not expected to change in a SW segment. However, occasionally a change in orientation is observed, typically at the beginning of the segment. This may happen if the MKF system reacts with some delay to a change in orientation associated with a turn. To remove the risk of these situations, we compute the mode of the orientations in each SW segments, and compare it with the mode of orientations in the previous SW segment for turn detection.

The orientation resolution (45° or 90°) to be chosen for the MKF orientation tracker depends on the specific environment. Although most corridor networks intersect at ±90°, in some cases intersections at multiples of ±45° should be accounted for. For example, in the WeAllWalk dataset, 13% of all turns are ±45° turns. Note that a change of orientation by 180° or (when using orientation resolution of 45°) by ±90°, is often detected as a close sequence of orientation changes by ±90° (or ±45°). This is usually not a problem in our two-stage strategy, where turns are declared only by comparing the orientation in two consecutive SW segments. For example, if a sequence of two 45° turns is detected during a non-SW segment, our system will declare that one 90° turn occurred (see Figure 2).

**Figure 4:**
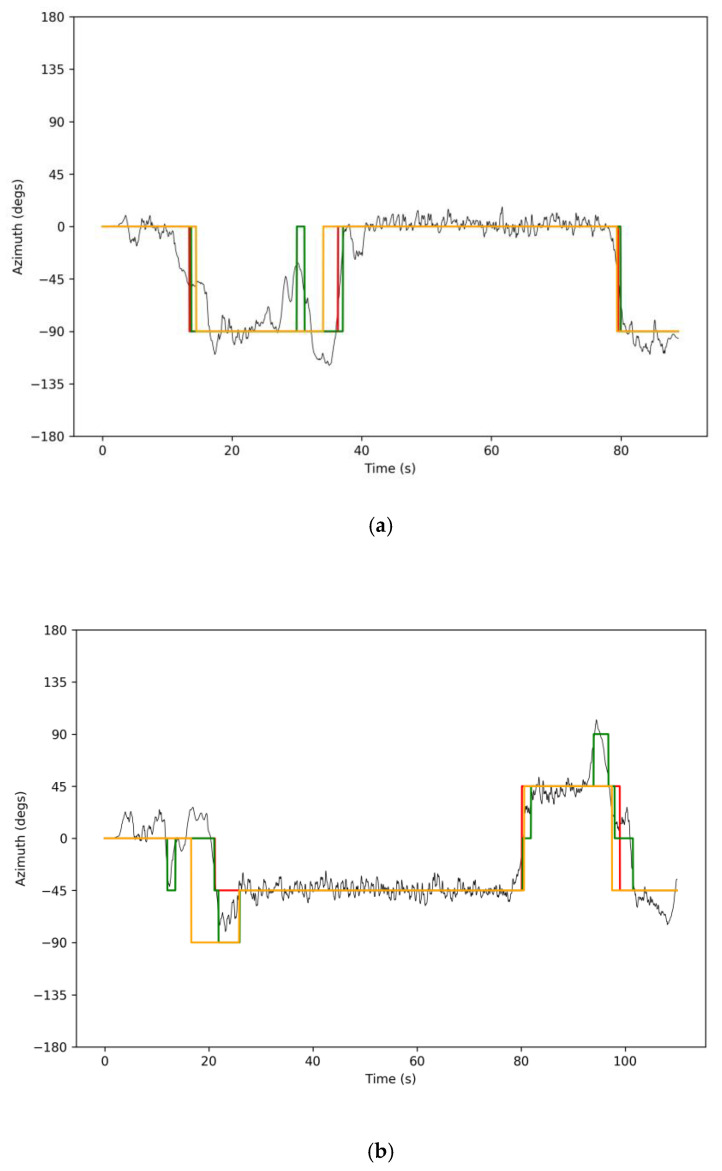
Example of two-stage turn detection. Black line: azimuth signal. Green line: walker’s discrete orientation as tracked by MKF. Orange line: walker’s discrete orientation as obtained by integrating the turns detected by the two-stage system. Red line: ground-truth walker’s discrete orientation. The orientation resolution of the MKF was 90°  (**a**) or 45° (**b**).

Sample results from our two-stage turn detection system are shown in Figure 4 (SW detection is not shown in these figures). Note that in Figure 4a, at around t = 30 s, a close sequence of orientation changes (left then right) as measured by the MKF was triggered as a consequence of the walker veering off the straight path (note the large variation in azimuth angle). As this happened outside of a SW period, these incorrectly detected turns were rejected by the two-stage system. A similar situation can be observed in Figure 4b, where the MKF was set to measure orientations at multiples of 45°.

### 3.3. RoNIN

RoNIN [10] is a deep network end-to-end odometry algorithm that was shown to outperform comparable systems in challenging datasets. It uses acceleration and gyro data, along with attitude data, to compute velocity vectors which, integrated over time, produce the user’s position. (We do not utilize the RoNIN body heading network since we are only interested in reconstructing trajectories). RoNIN uses a heading-agnostic reference frame. In practice, the 3-D vectors of acceleration and gyro are pre-multiplied by the inverse of the attitude matrix, so that they effectively are defined on a fixed “world” reference frame with z axis pointing in the direction of gravity. Although this reference frame normalization was shown to enable robust odometry, any drift accumulated in the computed attitude also reflects in the velocity vectors produced in output.

In order to apply RoNIN to the WeAllWalk inertial data, it is first necessary to up-sample (via linear interpolation) the data from the original acquisition rate of 25 Hz to 200 Hz, which is the rate at which sensor data were collected for RoNIN training. We used the open source implementation provided by the authors (https://github.com/Sachini/ronin; accessed on 9 June 2021) and chose the RoNIN resnet18 architecture. Since the data used for training RoNIN came from sensors that are different from those in the iPhones used in WeAllWalk, results may be different than expected. As a simple customization, we regressed a constant scaling factor to minimize the discrepancy between the magnitude of the velocities computed by RoNIN and of the ground-truth velocities in WeAllWalk. Least squares regression was used to compute an appropriate scaling factor (found to be equal to 1.27) to be applied to the velocity vectors produced by RoNIN.

We also considered a more extensive customization of RoNIN, by fine-tuning the network on WeAllWalk data. Note that RoNIN requires ground-truth values for the walker’s location and orientation at all times, defined with respect to the world reference frame used to define the phone attitude; whereas WeAllWalk only contains the times at which each walker crossed specific waypoints. To generate the missing data, we interpolated the walker’s location between straight segments’ waypoints assuming constant velocity, and assumed that, in a given segment, walkers maintained a constant orientation parallel to the corridor. In order to recover the walker’s orientation with respect to the world reference frame, we rely on the orientation estimated at each time by the original RoNIN network. We then fine-tuned the RoNIN network (resnet18 architecture) for two epochs, with minibatch size set to 128, using the Adam optimizer with learning rate of 0.0001. We only considered this customization for the blind walkers, given that the original RoNIN was already trained on sighted walkers. Indeed, we verified that fine-tuning the algorithm on the sighted walkers in WeAllWalk did not lead to noticeable improvements.

### 3.4. Particle Filtering

When a map of the environment is available, the constraint of wall impenetrability can benefit path reconstruction. Bayes filtering (in particular, particle filtering [9]) is typically used in these situations, as it can easily incorporate the wall impenetrability constraint by postulating an appropriate form for the joint distribution of the state s being tracked. For example, if the state contains the location of the walker, the joint probability $P\left(s\left(t\right),s\left(t+\Delta t\right)\right),\;$ where Δt is the sampling period, is set to be 0 if $s\left(t\right)$ and $s\left(t+\Delta t\right)$ identify two locations across a wall from each other. Particle filtering is a particular form of Bayes filtering wherein the posterior distribution of the state is represented by a set of samples (particles). Statistics such as the posterior mean can be obtained by simply computing the mean location of these particles.

In our implementation, the particle filter receives in input a sequence of velocity vectors $v\left(t\right)\;=||v\left(t\right)||\;\cdot \;{\left[\cos\theta \left(t\right),\sin\theta \left(t\right)\right]}^{T}$, which are generated either by the RoNIN algorithm (in its original or fine-tuned version, and subsampled to a rate of 25 Hz), or by forming a vector in the direction of the azimuth, as measured by the phone, with length equal to the stride length considered, divided by the duration of the current step, as measured by our step counter. The state being tracked has two components: the current location of the walker, $P_{t}$, and the current drift angle, $d_{t}$. State evolution is modeled as:

(2) $$P\left(t+\Delta t\right)\;=P\left(t\right)\;+\;\left(||v\left(t\right)||\;+\;n_{v}\right)\;\cdot \;{\left[\cos\left(\theta \left(t\right)\;+\;d\left(t\right)\;+\;n_{\theta }\right),\sin\left(\theta \left(t\right)\;+\;d\left(t\right)\;+\;n_{\theta }\right)\right]}^{T}\cdot \Delta t\;d\left(t+\Delta t\right)\;=d\left(t\right)\;+\;n_{d}$$

Here, $n_{v}$, $n_{\theta }$, and $n_{d}$ represent mutually uncorrelated zero-mean Gaussian white noise. We set the standard deviations of $n_{\theta }$ and $n_{d}$ to 0.01 rads and 0.005 rads, respectively, where these values were found empirically through preliminary tests. We noted that the system performance is rather sensitive to the choice of the standard deviation $\sigma_{v}$ of $n_{v}$, and, thus, decided to determine this value systematically through grid search on training data. We assume that the location of the starting point in the map is known, and perform an initial alignment procedure (similar to [10]) by matching the path reconstructed in the initial few seconds with the orientation of the corridor where the starting point is located. The particle filter uses 1000 particles.

**Figure 5:**
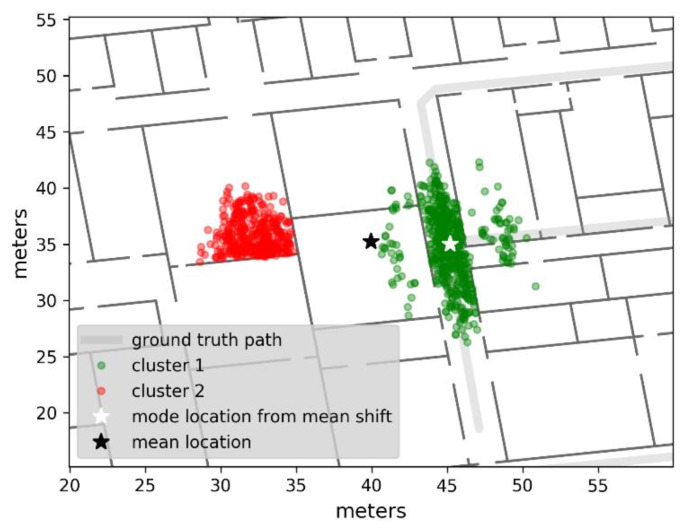
The set of particles at a certain time during tracking. Note that the particles are distributed in two main clusters (shown in different colors). The mean particle location (posterior mean), shown by a black star, is in an incorrect position. The highest mode of the distribution, as found by mean shift, is shown by a white star.

A problem with Bayes filtering as applied for path reconstruction is that it often produces bi-modal posterior state distributions (see Figure 5). In these situations, the posterior mean often falls in areas of low probability (in-between the modes). In practice, this means that the mean location of the particles may fall in a location that appears to break the wall non-penetrability constraint. Rather than the posterior mean, one could consider the modes (maximizers) of the posterior distribution, which are relatively immune to these artifacts. For example, Fusco and Coughlan [63] used a kernel density estimator to find the mode of the state posterior distribution. We took a different route and used the mean shift algorithm [74], a technique based on gradient ascent to find the modes of a distribution expressed as a set of samples. Our Particle Filtering–Mean Shift (*PF-MS*) algorithm thus estimates the walker’s location as the location of the highest mode identified by Mean Shift. If one is interested only in the reconstructions of the whole trajectory (and not in instantaneous location estimation), a variation of PF-MS can be used. Specifically, one can track the modes of the posterior state distribution, by associating a mode at time t+Δt with the mode at time t that shares the largest number of supporting particles. At the end of the trajectory, the highest mode of the final posterior distribution is found, then the chain of pairwise associated modes in the previous time instants is reconstructed. We named this “global” strategy *PF-MS-G*. We used the python mean shift implementation from sklearn, setting the bandwidth to 5 m, a value found empirically in preliminary tests.

The digital maps of the building walls were obtained by tracing the original maps provided as part of WeAllWalk using the SIM web application (https://sim.soe.ucsc.edu; accessed on 9 June 2021) [75]. We assumed that all doors in the buildings were open, meaning that the estimated paths could potentially go through such doors into rooms (even though the participants only walked along the buildings’ corridors.)

## 4. Experiments

### 4.1. Training and Test Modalities

The algorithms discussed in Section 3 contain a number of trainable parameters, and specifically: the parameters of the LSTM used for step counting, along with the threshold on the output of LSTM and the stride length (Section 3.1); the values for $\sigma_{\theta },\sigma_{d}$, and q in the MKF used for orientation tracking (Section 3.2.2); the parameters of the GRU used for SW detection (Section 3.2.1); and the value of $\sigma_{v}$ for the particle filter. In addition, as discussed in Section 3.3, the RoNIN algorithm was fine-tuned for the blind participants.

Since the walking characteristics of blind persons using a long cane or a dog guide are expected to be different [76], we stratified the results by community of long cane users (denoted by the modifier *:LC*) and dog guide users (*:DG*). We considered the following training and test schemes. Note that the system used to test any given walker was never trained on data from that walker.

- **Train on Sighted (TS).** All parameters are computed using data from the five sighted walkers in WeAllWalk. The system is tested on the two community of blind users (*TS:LC*, *TS:DG*). This may be representative of a situation in which a system designed for sighted walkers is used by blind walkers, without any customization.
- **Train in same Community (TC).** In this case, the system tested with long cane users, dog guide users, and sighted users, was trained with data from walkers in the same community. We used the *Leave-One-Person-Out* cross–validation policy [77]: each participant was tested with the system trained on data from all other participants in the same community. This training modality allows us to test the hypothesis that walking characteristics may be different between communities of users. If this hypothesis is true, one may expect that training the system on the same community of users who are meant to use it should give improved results. In addition to the two communities of blind participants (*TC:LC*, *TC:DG*), for this modality only we also present results for the sighted participants (*TC:S*). The latter quantity may be representative of a conventional system, trained and tested on sighted users, and can be used as a benchmark. Of note, only three walkers in WeAllWAlk used a dog guide, and, thus, each training set in the *TC:DG* modality contain data from two walkers only.
- **Train on All (TA).** All available data in WeAllWalk were used for training, using the Leave-One-Person-Out cross–validation policy (*TA:LC*, *TA:DG*). For example, a long cane user is tested with a system trained with data from all sighted participants, all dog cane users, and all other long cane users.

For all tests in each modality, the measured quantities of interest are averaged over both iPhones carried by the participants, all paths, and all participants in the test set.

### 4.2. Step Counting

**Figure 6:**
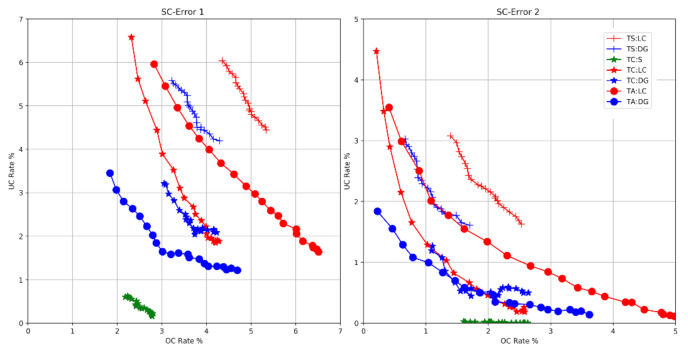
UC rate vs. OC rate curves as a function of the threshold S on the LSTM output.

We considered two different error metrics for the step counter. The first metric (*SC-Error 1*) is a variation of the metric with the same name in [16]. We compute the midpoint between any two consecutive ground-truth heel strike times, and count the number of detected steps between two consecutive such midpoints. Note that there is exactly one ground-truth heel strike within this interval. If n>1 steps are detected within this interval, n−1 *overcount* events are recorded. If no steps are detected, an *undercount* event is recorded. For the second metric, SC-Error 2 [16], the difference between the number of detected steps in a WeAllWalk segment and the number of ground-truth heel strikes in the same segment is recorded as overcount (if positive) or undercount (if negative). The total number of overcount and undercount events in each path is normalized by the total number of ground-truth heel strikes to produce an undercount (UC) rate and an overcount (OC) rate. Note that increasing the threshold S on the output of the LSTM (Section 3.1) normally results in an increase in the UC rate and a decrease in the OC rate. This is shown in Figure 6, where we plotted the UC rate vs. OC rate as a function of S.  For the test results shown in Table 1, the threshold S is set to a value that equates the OC and UC rates of SC-Error 2 measured in the training data. The values of S thus computed, averaged over all cross-validation rounds, are also shown in Table 1, along with the average value of stride length SL regressed from the training data as discussed in Section 3.1.1.

### 4.3. Turn Detection

To quantitatively assess the performance of our two-stage turn detector (Section 3.2), we compare the sequence of detected turns against the ground-truth sequence of turns in each path using Levenshtein distance. Specifically, we find the longest ordered matching subsequences within the two turns sequences, then report errors in terms of overcounts (detected turns that were not matched in the ground-truth sequence) and undercounts (ground-truth turns that were not matched in the detected sequence). Note that, in the case of a 45° ground-truth turn, a 90° turn detector would either not detect the turn, or detect it as a 90° turn, where both cases represent an incorrect result. To simplify analysis of the results, we simply removed the 45° turns and any associated 90° turn detection from the analysis of the 90° turn detector. When assessing the 45° turn detector, we split all ground-truth 90° turns as well as all measured 90° turns into two consecutive 45° turns. The number of turn undercounts and overcounts was divided by the total number of ground-truth turns to obtain the undercount (UC) and overcount (OC) rates, which together define the turn detection (*TD*) error. Results are shown in Table 2.

### 4.4. Path Reconstruction

#### 4.4.1. Evaluation Metrics

Each reconstructed trajectory is compared with the ground-truth path. WeAllWalk does not provide detailed information of the walkers’ location at each time, but only the timestamps $\left\{t_{j}^{i}\right\}$ indicating when a walker reached individual waypoints. We will make the simplifying assumption that walkers were located in the middle of the corridor width when transitioning between segments through each such waypoint.

Given a trajectory estimated for a walker in a path, $P^{i}\left(t\right)$, we use the locations at the waypoint timestamps, $\left\{P^{i}\left(t_{j}^{i}\right)\right\}$ to first *align* this trajectory with the ground-truth path. This standard procedure [78] is necessary because the reference frame used to represent the trajectory is undefined. Specifically, we find (using Procrustes analysis) the rotation and translation that minimizes the mean squared distance between the locations $\left\{{\bar{P}}_{j}^{i}\right\}$ (the ground-truth location of the waypoints) and $\left\{P^{i}\left(t_{j}^{i}\right)\right\}$. After alignment, we evaluate the goodness of the estimated path using three different metrics. The first metric considered is the RMSE of estimated waypoint locations:

(3) $${\mathrm{RMSE}}_{\mathrm{wp}}=\sqrt{\frac{1}{N}\sum_{j}||{\bar{P}}_{j}^{i}-P^{i}{\left(t_{j}^{i}\right)||}^{2}}$$

where N is the number of waypoints in the path. For the remaining metrics, we consider a sampling of the estimated trajectory into $N_{e}^{i}$ points $\left\{Q_{m}^{i}\right\}$ with a uniform inter-sample distance of 1 m. Likewise, we sample the segments joining consecutive waypoints at intervals of 1 m, resulting in $N_{gt}^{i}$ points $\left\{{\bar{Q}}_{n}^{i}\right\}$ representing the path. We then compute the Haussdorff distance between these two sets of points, as well as their (weighted) average Haussdorff distance [79]:

(4) $$\mathrm{Hauss}=\max\left(\underset{m}{\max}\left(\underset{n}{\min}\left(||Q_{m}^{i}-{\bar{Q}}_{n}^{i}||\right)\right),\underset{n}{\max}\left(\underset{m}{\min}\left(||Q_{m}^{i}-{\bar{Q}}_{n}^{i}||\right)\right)\right)$$

(5) $$\mathrm{avHauss}=\frac{1}{2}\left(\sqrt{\frac{1}{N_{e}}\sum_{m}\underset{n}{\min}\left(||Q_{m}^{i}-{\bar{Q}}_{n}^{i}||{}^{2}\right)}+\sqrt{\frac{1}{N_{\mathrm{gt}}}\sum_{n}\underset{m}{\min}\left(||Q_{m}^{i}-{\bar{Q}}_{n}^{i}||{}^{2}\right)}\right)$$

**Figure 7:**
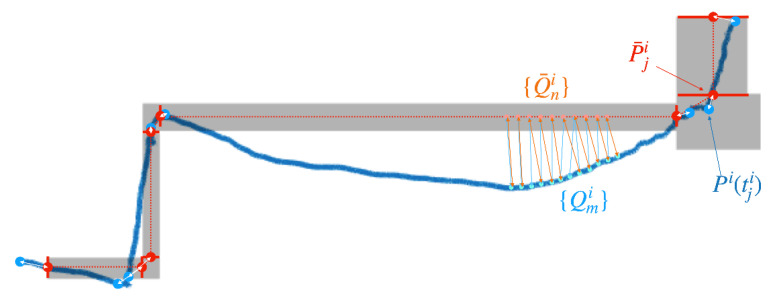
An illustration explaining the metrics used to evaluate the quality of an estimated trajectory $P^{i}\left(t\right)$ (shown as blue line after alignment). The gray shape shows the location of the corridors along the path, with individual segment separation shown in red. Waypoints (${\bar{P}}_{j}^{i}$) are shown as red dots, while the estimated walker’s locations at waypoint timestamps ($P^{i}\left(t_{j}^{i}\right)$) are shown as blue dots. The distances $||{\bar{P}}_{j}^{i}-P^{i}\left(t_{j}^{i}\right)||$ are shown by white arrows. The uniform distance sampling of the segments joining consecutive waypoints ($\left\{{\bar{Q}}_{n}^{i}\right\}$) is shown by orange dots, while that of the estimated trajectory ($\left\{Q_{m}^{i}\right\}$) is shown by light blue dots. The associated distances $\underset{m}{\min}\left(||Q_{m}^{i}-{\bar{Q}}_{n}^{i}||\right)$ and $\underset{n}{\min}\left(||Q_{m}^{i}-{\bar{Q}}_{n}^{i}||\right)$ are shown with orange and light blue arrows, respectively.

The Haussdorff distance penalizes any large (possibly episodic) discrepancy between the estimated trajectory and the ground truth. The average Haussdorff is a more lenient measure, that penalizes consistent biases. These two metrics allow us to evaluate the goodness of the estimated trajectory in its entirety (and not just at waypoints). Figure 7 illustrates the three considered metrics.

#### 4.4.2. Map-Less Path Reconstruction

**Figure 8:**
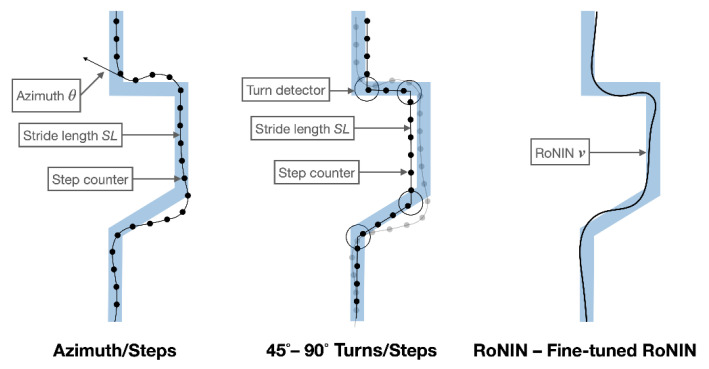
Diagrammatic examples of the algorithms used for map-less path reconstruction. The blue line represents the path taken by the walker. The black line represents the estimated path. Dots represent heel strikes; circles represent turns.

We considered the following algorithms for map-less path reconstruction (see Figure 8):

- **Azimuth/Steps (A/S)**: At each detected step, a displacement vector is defined with length equal to the step stride, and with orientation equal to the azimuth angle θ as provided by the phone;
- 45°**–**90°**Turns/Steps (T/S)**: At each detected step, a displacement vector is defined with length equal to the step stride, and with orientation equal to the output of our two-stage 45° or 90° turn detection method;
- **RoNIN (R)–Fine-tuned RoNIN (FR)** (see Section 3.3).

**Figure 9:**
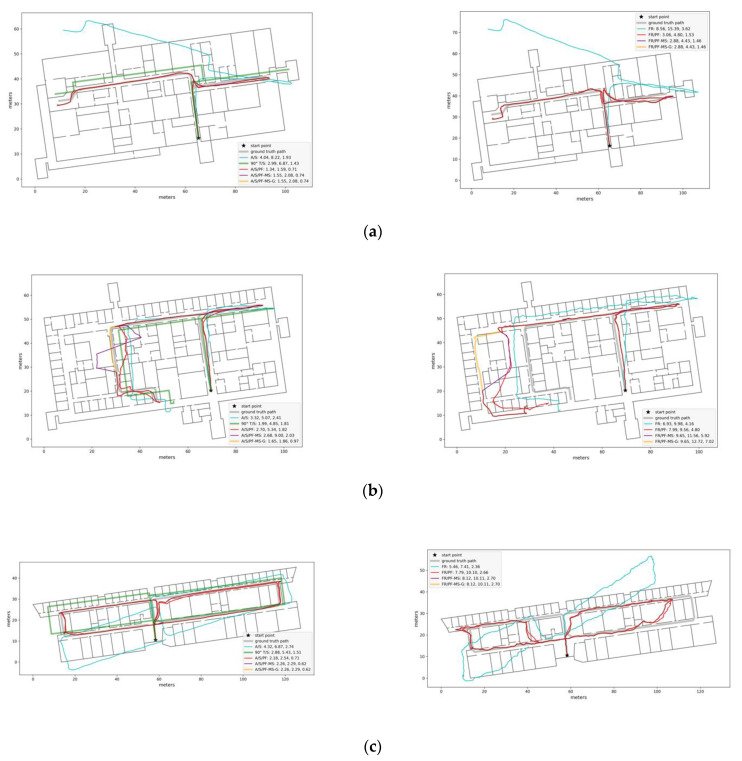
Examples of path reconstruction from the TA:LC training/test modality. Left: Map-less: A/S, 90° T/S; Map-assisted: A/S-PF, A/S-PF-MS, A/S-PF-MS-G. Right: Map-less: FR; Map-assisted: FR-PF, FR-PF-MS, FR-PF-MS-G. Reconstruction errors for the three metrics (RMSEwp, Hauss, avHauss) are shown (in units of meters) in the legend of each figure. (**a**–**c**) refer to different buildings.

Reconstruction errors are shown for the three considered metrics in Table 3. Note that fine-tuned RoNIN was only considered for blind walkers (LC and DG). Examples of map-less path reconstruction using fine-tuned RoNIN, Azimuth/Steps, and 90^o^ Turns/Steps, are shown in Figure 9. Although the reconstruction errors are computed after alignment as described in Section 4.4.1, the paths have been re-aligned on the underlying map in the figure for easy comparison with the map-assisted reconstructed paths.

#### 4.4.3. Map-Assisted Path Reconstruction

We experimented with feeding the particle filter with data generated by three different algorithms: Azimuth/Steps (A/S), RoNIN, and, for blind walkers, fine-tuned RoNIN. The Turns/Steps algorithm was shown to give comparatively poor results in this case. Table 4 shows the results, for the three metrics considered, using particle filtering (PF), as well as particle filtering with mean shift mode selection in the “instantaneous” mode (PF-MS) and in the “global” mode (PF-MS-G; see Section 3.4). Sample reconstructed paths are shown in Figure 9.

## 5. Discussion

### 5.1. Step Counting

The data in Table 1 and the curves in Figure 6 clearly show how step counting accuracy is affected by the community of walkers used to train the system. For example, when testing with long cane users a step counter trained on sighted walkers (TS:LC), the sum of undercount and overcount rates was found to be 13.09% (SC-Error 1) or 8.33% (SC-Error 2). However, when the system was trained only with other long cane users (TC:LC), these numbers reduce to 6.49% and 1.87%, respectively. Similar observations can be drawn from the tests with dog guide users, for whom the best results were obtained when training on all available data (TA:DG). A possible cause for the worse performance of TC:DG with respect to TA:DG is the small number of dog guide users in WeAllWalk (only two users in the training set of each cross-validation round).

The average threshold S on the output of the LSTM as learned from within-community training data is substantially larger for sighted walkers (0.78) than for dog guide (0.68) or long cane users (0.55). Larger thresholds should be expected when the output of the LSTM is closer to the binary signal used to indicate heel strikes. This suggests that the LSTM is better capable of modeling the desired output for sighted walkers (possibly due to their more regular gait) than for blind walkers. The average stride lengths learnt within-community are also larger for sighted walkers (0.74 m) than for dog guide users (0.62 m) or long cane users (0.55 m). This is not surprising considering that, in general, dog guide users walk faster and more confidently than long cane users, as they do not need to probe the space ahead with the cane and can rely on their dog guide to lead them along a safe trajectory. Comparison with [16], which also presents results for various step counting algorithms as applied to WeAllWalk, shows that use of a LSTM leads to improved results. For example, the lowest value for SC-Error 1 (measured as the sum of UC and OC rates) for long cane users was found to be 7.8% in [16] (vs. 6.5% with our system, see Table 1). For the same community of users, the minimum SC-Error 2 found in [16] was 4.8%, vs. 1.9% with our system.

### 5.2. Turn Detection

Remarkably, Table 2 shows no undercounts or overcounts for sighted walkers (TC:S). This suggests that these participants tended to walk on straight lines, without large sway patterns that could generate false positives. Errors were generally higher for the 45° than for the 90° turn detectors. These results should be evaluated keeping in mind that even a single missed turn, or a single false positive, could potentially lead to large path reconstruction errors. For long cane users, training with all available data (TA:LC) gave substantially better results than training only with data from sighted walkers (TS:LC). No such large discrepancies across training modalities were observed when testing with dog guide users, for whom the best results were obtained for within-community training (TC:DG). These results are vastly superior than those observed in [16], where turns were computed on WeAllWalk data using the algorithm described in [38]. In that case, the accumulated error (UC rate + OC rate) 90° TD-Error was found to exceed 50%.

### 5.3. Map-Less Path Reconstruction

The data in Table 3 shows that the smallest reconstruction errors were measured for the 90° Turns/Steps algorithm (although for dog guide users, a similar small error for the avHuass metric was obtained with fine-tuned RoNIN). The 45° Turns/Steps algorithm performed only marginally worse than the 90° case. Remarkably, the best training modality for long cane users (within-community training, TC:LC), gives similar error as the best case for sighted walkers. However, when testing with long cane user using a system trained with sighted walkers, very poor results were obtained. For example, the RMSE_wp_ error for the 90° Turns/Steps system, which is 3.46 m for within-community training (TC:LC), jumps to 9.03 m when training on sighted walkers (TS:LC). For dog guide users, the best results were obtained by training over all available data. Both RoNIN and the simpler Azimuth/Steps algorithms are affected by drift, and produced comparable results.

One notable exception is for long cane users when the system was trained with sighted walkers (TS:LC). In this case, RoNIN gave substantially better results than all other methods (though still worse than TC:LC). A likely reason for this can be found in the different average stride length between sighted and long cane users (see Section 5.1), which may cause incorrect reconstructed path lengths for TS:LC. RoNIN, which does not rely on stride lengths, may provide more accurate velocity measurements in this case. The data also shows that fine-tuning the RoNIN network did not result in improved path reconstruction performance.

### 5.4. Map-Assisted Path Reconstruction

The best results for the community of blind walkers were obtained with the azimuth/steps (A/S) algorithm, processed by the PF-MS-G filter. For the sighted walkers, the best results were obtained with RoNIN, still processed by PF-MS-G, although these last results were only marginally better than using the A/S algorithm. It appears that the strong wall impenetrability constraint was able to limit the effect of azimuth drift. In general, errors for map-assisted reconstruction were substantially lower than for the map-less case. As in the prior cases, training the system over sighted walkers was shown to give poor results when tested with long cane users and, to a lesser extent, with dog guide users. Although the best results were obtained with the PF-MS-G algorithm, use of the mean shift clustering was not shown to give consistently better results overall.

Figure 9 provides some insight into the behavior of the reconstruction algorithms. In Figure 9a, both original paths (A/S, fine-tuned RoNIN) were grossly incorrect due to orientation drift. Particle filtering tracked and removed drift, and correctly reconstructed the paths. In the case of Figure 9b, poor velocity estimation led to reconstructed segments that were too short (Azimuth/Steps) or too long (fine-tuned RoNIN). In both cases, particle filtering found incorrect paths through open doors. PF-MF-G was able to correctly reconstruct most of the path for the Azimuth/Steps case, but not for the fine-tuned RoNIN case. The trajectory in output of either system in the case of Figure 9c was affected by both drift and incorrect velocity estimation. Although particle filtering was for the most part able to successfully correct the trajectory in the Azimuth/Steps case, it produced a poor reconstruction for fine-tuned RoNIN.

We note that the set of path reconstruction algorithms considered in this work includes inertial-based odometry algorithms used in prior research for blind walkers. For example, [68] used a PDR-based on step counting coupled with orientation (equivalent to our A/S system), while [67] additionally implemented a particle filter (equivalent to our A/S-PF system).

## 6. Conclusions

We have presented a detailed study on the use of two odometry systems: a simple PDR based on step counts and orientation estimation, and a deep learning algorithm (RoNIN), to reconstruct paths taken by blind walkers using a long cane or a dog guide, as represented in the WeAllWalk dataset. We have considered both the map-less case and the map-assisted case. For the map-less case, we have introduced a two-stage system capable of robustly detecting turns at multiples of 45° or 90° degrees, combined with an RNN-based step counter with learned fixed stride length. For the map-assisted case, we employed a standard particle filtering algorithm, with the addition of a posterior distribution mode identification module. Compared with prior work that explored inertial odometry for use by blind pedestrians (e.g., [18,67,68]), our study includes a variety of algorithms for both the map-assisted and map-less case, and reports the results of extensive quantitative evaluations using appropriate metrics on a representative data set with blind walkers (WeAllWalk). Although it is possible that other sensing modalities (e.g., visual odometry [63] or BLE-based positioning [65]) may achieve higher localization accuracy, inertial odometry has clear practical advantages, as it requires no additional infrastructure, and may function even if the user keeps the smartphone in their pocket while walking.

Our study has shown that, for the same algorithm, the choice of the community of walkers used for training the algorithm’s parameters is critical. Systems trained with sighted walkers consistently gave poor results when tested with long cane users and, to a lesser extent, with dog guide users. However, when the training set contained data collected from these communities, results improved substantially, and were in fact comparable to the best results obtained when testing with sighted walkers. Our results also showed that our simple Turns/Steps PDR produced better results than the more sophisticated RoNIN in the map-less case, even when the latter was fine-tuned to better model walking patterns of blind individuals. For the map-assisted case, the best results were found when the particle filter was fed with data from the simple Azimuth/Steps algorithm. It should be noted, however, that participants in WeAllWalk kept their smartphones in a fixed location on their garments. Had they changed the orientation of their phone (e.g., to pick up a call, or by repositioning the phone in a different pocket), it is likely that this change in phone orientation would have negatively affected the results. In these situations, an algorithm such as RoNIN, which was trained to correctly identify the user velocity independently of the phone orientation, possibly combined with a mechanism to reduce the effect of drift, could provide more robust position tracking.

The choice of the minimum turn angle for a Turn/Steps PDR depends on the specific environment considered. Although the layout of most buildings is made by corridor networks intersecting at 45° or 90° degrees, there may be situations that call for a finer angular interval. Although, in principle, this could be achieved simply by increasing the cardinality of the state tracked by the MKF (Section 3.2.2), in practice this could result in increased false positives. In addition, we have found (as mentioned in Section 3.2.3) that turns of 90° degrees are often detected as a sequence of two 45° turns. We expect that a similar behavior would be amplified in the case of a finer angular resolution.

## Figures and Tables

**Table 1:** Undercount (UC) and overcount (OC) rates for our step counter, along with the mean threshold S and stride length SL. For each community of blind walkers (LC, DG), the pair (UC rate, OC rate) with the smallest value of their sum is shown in boldface.

	SE-Error 1		SE-Error 2		Mean S	MeanSL (m)
	UC Rate %	OC Rate %	UC Rate %	OC Rate %		
TS:LC	10.58	2.51	8.20	0.13	0.78	0.74
TS:DG	8.74	2.08	6.75	0.10	0.78	0.74
TC:S	3.03	1.05	2.24	0.26	0.78	0.74
TC:LC	**3.22**	**3.27**	**0.91**	**0.96**	0.55	0.55
TC:DG	4.99	2.15	3.23	0.39	0.68	0.62
TA:LC	4.06	3.80	1.42	1.17	0.56	0.62
TA:DG	**2.29**	**2.55**	**0.83**	**1.08**	0.55	0.62

**Table 2:** Undercount (UC) and overcount (OC) rates for our turn detector (45° and 90° turns). For each community of blind walkers (LC, DG), the pair (UC rate, OC rate) with the smallest value of their sum is shown in boldface.

	45° TD-Error		90° TD-Error	
	UC Rate %	OC Rate %	UC Rate %	OC Rate %
TS:LC	0.64	6.85	0	1.87
TS:DG	1.14	4.49	0.81	0.81
TC:S	0	0	0	0
TC:LC	1.64	3.98	0.54	0.26
TC:DG	**1.11**	**4.30**	**0**	**0.79**
TA:LC	**0.37**	**3.51**	**0**	**0.79**
TA:DG	1.15	5.39	0	0.83

**Table 3:** Reconstruction errors (*RMSE_wp_, Hauss, avHauss*) using the map-less path reconstruction algorithms described in Section 4.4.2. Units of meters. For each community of walkers (S, LC, DG), the smallest error values for each metric are shown in boldface.

	A/S			45° T/S			90° T/S			R			FR		
TS:LC	9.43	14.68	4.90	9.17	14.48	4.70	9.03	13.90	4.63	5.43	8.56	2.93	–	–	–
TS:DG	5.81	8.97	3.30	5.64	8.67	2.82	4.94	**7.50**	2.75	5.66	8.55	2.84	–	–	–
TC:S	4.45	7.06	2.39	3.96	6.12	1.89	**3.93**	**5.97**	**1.88**	4.26	6.75	2.39	–	–	–
TC:LC	3.85	6.21	2.30	3.86	6.45	2.22	**3.46**	**5.47**	**1.97**	5.43	8.56	2.93	4.36	7.37	2.54
TC:DG	6.38	9.90	3.29	6.28	9.86	2.92	6.13	9.60	2.92	5.66	8.55	2.84	6.80	10.42	3.29
TA:LC	6.29	10.27	3.60	5.99	9.79	3.32	5.88	9.47	3.31	5.43	8.56	2.93	6.18	9.90	3.27
TA:DG	5.21	8.37	2.88	5.00	8.18	2.52	**4.59**	7.64	**2.50**	5.66	8.55	2.84	5.17	8.08	**2.50**

**Table 4:** Reconstruction errors (*RMSE_wp_, Hauss, avHauss*) using the map-assisted path reconstruction algorithms described in Section 4.4.3. Units of meters. For each community of walkers (S, LC, DG), the smallest error values for each metric are shown in boldface.

	A/S PF			A/S PF-MS			A/S PF-MS-G			R PF			R PF-MS			R PF-MS-G			FR PF			FR PF-MS			FR PF-MS-G		
TS:LC	5.68	8.11	2.62	5.98	9.48	2.80	5.53	8.40	2.44	4.80	7.01	2.47	5.01	7.72	2.54	4.83	6.90	2.28	-	-	-	-	-	-	-	-	-
TS:DG	4.07	5.78	1.78	4.15	6.36	1.81	3.87	5.86	1.62	5.19	7.45	2.48	5.28	7.61	2.46	5.25	7.33	2.33	-	-	-	-	-	-	-	-	-
TC:S	3.35	4.93	1.60	3.35	5.01	1.48	3.32	4.80	1.38	3.10	4.46	1.46	3.29	4.92	1.45	**2.96**	**4.24**	**1.19**	-	-	-	-	-	-	-	-	-
TC:LC	2.78	3.95	1.36	2.86	4.35	1.28	**2.73**	**3.49**	**1.08**	5.05	7.44	2.54	5.17	7.98	2.51	4.95	7.37	2.30	3.54	5.43	1.89	3.62	6.15	1.86	3.50	5.24	1.66
TC:DG	5.77	8.12	2.46	6.09	8.50	2.61	6.08	8.39	2.35	5.56	7.98	2.74	6.08	8.65	2.92	5.47	7.64	2.39	6.10	8.68	3.03	6.19	9.62	2.97	6.16	8.59	2.77
TA:LC	3.30	4.71	1.62	3.46	6.21	1.68	3.20	5.06	1.44	5.36	7.69	2.54	5.55	8.38	2.62	5.40	7.69	2.40	4.53	6.53	2.32	4.79	7.29	2.42	4.51	6.55	2.13
TA:DG	4.19	6.10	1.99	4.62	6.44	2.03	**3.80**	**5.13**	**1.54**	5.09	7.56	2.48	5.29	8.13	2.46	5.23	7.62	2.40	4.28	6.01	2.16	4.32	6.64	2.14	3.97	5.56	1.81

## Data Availability

Publicly available datasets were analyzed in this study. This data can be found here: [https://datadryad.org/stash/dataset/doi:10.7291/D17P46, accessed on 9 June 2021].
